# Prognostic nutritional index as a biomarker for identifying coronary artery lesions in Kawasaki disease

**DOI:** 10.1016/j.jped.2025.101436

**Published:** 2025-08-18

**Authors:** Chao Zhang, Guoshun Mao, Guosheng Hu

**Affiliations:** a Fu Yang People's Hospital, Department of Pediatrics, Fuyang, China; b Fu Yang People's Hospital of Anhui Medical University, Fuyang, China

**Keywords:** Kawasaki disease, Nutritional status, Coronary artery disease, Biomarkers, Prognostic nutritional index

## Abstract

**Objective:**

To evaluate the Prognostic Nutritional Index as a biomarker for identifying coronary artery lesions in Kawasaki disease.

**Methods:**

The clinicopathological and laboratory characteristics of 241 patients with Kawasaki disease were collected from patients who were hospitalized in Fuyang People's Hospital from January 2018 to December 2024. Logistic regression analysis was conducted to evaluate the relationship between Prognostic Nutritional Index and coronary artery lesions. The critical levels of hematological parameters were determined by receiver operating characteristic analysis.

**Results:**

The incidence of coronary artery lesions was 10.3 %. The optimal cut-off point for the Prognostic Nutritional Index was defined as 46.575. Prognostic Nutritional Index was negatively correlated with coronary artery diameter (*r* = −0.260, *p* < 0.001). Patients with low Prognostic Nutritional Index levels (Prognostic Nutritional Index < 46.575) were 4.25 times more likely to have coronary artery lesions compared to those with high Prognostic Nutritional Index levels (Prognostic Nutritional Index ≥ 46.575) (OR = 4.25, 95 % CI: 1.688 - 10.697, *p* < 0.001). The area under the receiver operating characteristic curve for Prognostic Nutritional Index in predicting coronary artery lesions was 0.702 (95 % CI: 0.584 - 0.821, *p* = 0.001), indicating moderate diagnostic efficacy.

**Conclusion:**

Prognostic Nutritional Index may be a biomarker for children with Kawasaki disease complicated with coronary artery lesions, and it may help identify high-risk patients of coronary artery lesions among children with Kawasaki disease.

## Introduction

Kawasaki disease (KD) is an acute febrile vasculitis of unknown etiology, predominantly affecting Asian children under 5 years of age. It has become the leading cause of acquired heart disease in children in developed countries, with 10–25 % of untreated KD patients developing coronary artery lesions (CALs) that may progress to severe complications such as coronary aneurysms, acute myocardial infarction, and sudden death [1,2]. Early assess and treatment of CALs to prevent coronary thrombosis are crucial in the acute phase of KD [3]. Accurate assessment of CALs is essential for clinical management in acute KD[4]. Biomarkers are vital for monitoring disease progression and therapeutic response, aiding in precise management. They also provide insights into the pathophysiology of KD-associated coronary artery complications, guiding future treatment strategies [5].

The prognostic nutritional index (PNI) is an indicator that combines serum albumin levels and peripheral blood lymphocyte counts, and it can reflect the nutritional and immune status of patients [6, 7, 8]. Low PNI levels are regarded as independent predictors of long-term mortality and major adverse cardiovascular events in patients with coronary heart disease [9,10]. In KD, PNI on the sixth day of fever is a reliable predictor of cardiovascular complications and intravenous immunoglobulin (IVIG) resistance [11]. Moreover, a PNI ≤ 40 is associated with longer persistence of coronary artery aneurysms over two years [12]. However, the correlation between PNI and CALs in KD remains underexplored. In this study, the authors retrospectively analyzed clinical data from 241 KD patients to evaluate PNI as a predictive biomarker for assess of CALs.

## Method

### Study design and population

This retrospective single-center study was conducted at Fu Yang People’s Hospital (Fuyang, China) and included 241 patients diagnosed with Kawasaki disease (KD) between January 2018 and December 2024. The study followed the diagnostic criteria for KD and coronary artery lesions (CALs) as specified by the American Heart Association (AHA) guidelines [2]. Inclusion Criteria: (1) Patients diagnosed with Kawasaki Disease (KD) according to the diagnostic criteria of the American Heart Association (AHA), including both complete and incomplete KD. (2) Patients aged under 18 years. Exclusion Criteria: (1) Other Chronic Diseases: Patients diagnosed with other chronic diseases (such as congenital heart disease, severe infections, autoimmune diseases, etc.). (2) Oncological Treatments: Patients undergoing oncological treatments (such as chemotherapy or radiotherapy). (3) Incomplete Data: Patients lacking necessary clinical data (such as blood test results for PNI calculation or echocardiography results). Based on echocardiographic findings at admission before treatment, the 241 KD patients were divided into two groups: the CALs group and the non-CALs group. The study protocol was approved by the Ethics Committee of Fuyang People’s Hospital (No. [2022]73). Written informed consent was obtained from all patients or their legal guardians before the use of their medical records in this study. The study was conducted in accordance with the Declaration of Helsinki.

### Data collection

This was a purely observational study on patients with KD, without any intervention. In this study, data were extracted from electronic medical records on December 6, 2024. The laboratory data consisted of hematological parameters required for the calculation of the Prognostic Nutritional Index (PNI). These parameters were derived from blood samples collected at the time of hospital admission, which corresponded to the 4.48 ± 2.25 days following the onset of the patients' fever symptoms. The parameters included white blood cell count, neutrophil count, lymphocyte count, hemoglobin concentration, platelet count, levels of aspartate aminotransferase (AST), alanine aminotransferase (ALT), total bilirubin, serum total protein, serum albumin, and C-reactive protein (CRP) concentration. Imaging data included echocardiography performed at admission.

Routine blood tests were performed using the Mindray 7500C automated blood cell analyzer (Shenzhen, China), and biochemical tests were conducted using the Siemens ADVIA 2400 automatic biochemical analyzer (Shanghai, China). The PNI was calculated using the formula: serum albumin (g/L) + 5 × total lymphocyte count (10^9/L).

The diagnosis of coronary artery lesions (CALs) was based on the Z-score method recommended by the American Heart Association (AHA) guidelines. The Z-score for coronary artery diameter was calculated using the Boston Z-score model, which adjusts for age, sex, and body surface area to provide a standardized measure of coronary artery size. A Z-score ≥2.0 is considered indicative of CALs [13,14].

### Statistical methods

Data were analyzed using SPSS version 22.0. The distribution of continuous variables was assessed using the Kolmogorov-Smirnov test. Normally distributed data were expressed as mean ± standard deviation (SD) and compared between groups using one-way analysis of variance (ANOVA). Non-normally distributed data were expressed as median and interquartile range (IQR) and compared using the Mann-Whitney U test. Categorical variables were described using frequencies and proportions, and differences between groups were assessed using the chi-square (χ^2^) test. The presence of coronary artery lesions (CALs) was assessed based on the American Heart Association (AHA) guidelines, which served as the gold standard. The diagnostic utility of the Prognostic Nutritional Index (PNI) was evaluated using receiver operating characteristic (ROC) curve analysis, with the area under the ROC curve (AUC) indicating the predictive accuracy. The optimal cut-off value for PNI was determined using Youden's index, which maximizes the sum of sensitivity and specificity. Patients were categorized into high and low PNI groups based on this threshold. Logistic regression analysis with forward stepwise variable selection was employed to identify independent risk factors for CALs. Variables with a p-value < 0.25 in univariate analyses were included in the multivariate model. A p-value of <0.05 was considered statistically significant in all analyses.

## Results

### Patient characteristics

Initially, 297 cases were screened for inclusion. Of these, 49 cases were excluded based on predefined exclusion criteria, and an additional 7 cases were excluded due to insufficient clinical data. The final sample comprised 241 patients. Within these patients, 135 were male (56 %) and 106 were female (44 %). The age range was from 1.5 to 72 months, with a mean age of 22.16 ± 16.14 months. Among the 241 children, 25 (10 %) were diagnosed with Coronary Artery Lesions (CALs). Children with CALs were significantly older compared to those without CALs (*p* < 0.05). Biochemical analyses revealed that children with CALs had significantly lower lymphocyte counts, serum albumin levels, and Prognostic Nutritional Index (PNI) values compared to those without CALs (*p* < 0.05). However, there were no significant differences between the two groups in terms of gender, rate of incomplete KD, hemoglobin, white blood cell count, neutrophil count, total bilirubin, alanine aminotransferase (ALT), aspartate aminotransferase (AST), erythrocyte sedimentation rate (ESR), C-reactive protein (CRP), platelet count, and total protein levels. The sociodemographic data and clinical characteristics of the patients are summarized in Table 1.

**Table 1:** Sociodemographic and clinical characteristics.

Clinical characteristics:	Total (*n* = 241)	Non-CALs group (*n* = 216)	CALs group (*n* = 25)	*F/χ^2^/Z* value	*P* value
Age (months)	22.16 ± 16.14	21.44 ± 16.04	28.36 ± 16.02	−2.425^b^	0.015
Gender, n ( %)				0.001	0.982
Female	107(44)	95(44)	11(44)		
Male	135(56)	121(56)	14(56)		
Height (m)	0.84 ± 0.14	0.83 ± 0.14	0.91 ± 0.13	−2.516 b	0.012
Weight (kg)	12.43 ± 3.93	12.29 ± 3.96	13.68 ± 3.47	−2.009 b	0.045
BMI (kg/m²)	17.41 ± 2.34	17.49 ± 2.27	16.73 ± 2.86	1.541 b	0.125
Duration of illness (days)	4.51 ± 2.26	4.40 ± 2.20	5.40 ± 2.53	−1.941^b^	0.052
Laboratory data at diagnosis					
Internal coronary artery diameter (mm)	2.17 ± 0.39	2.05 ± 0.19	3.17 ± 0.32	−10.832 b	<0.001
Hemoglobin (g/L)	105.25 ± 10.35	105.54 ± 10.38	102.72 ± 9.90	1.671 a	0.197
Leukocytes (× 10^9^/L)	14.76 ± 5.54	14.75 ± 5.36	14.77 ± 7.03	−0.157 b	0.875
Neutrophils (× 10^9^/L)	9.94 ± 7.32	9.52 ± 4.68	13.55 ± 18.01	−0.742^b^	0.458
Lymphocytes (× 10^9^/L)	3.83 ± 1.88	3.92 ± 1.90	3.00 ± 1.49	2.233^b^	0.026
Total bilirubin (μmol /L)	9.04 ± 8.98	8.84 ± 8.26	10.83 ± 13.97	−0.708 b	0.479
ALT (U/L)	91.93 ± 145.91	88.02 ± 131.98	126.34 ± 237.53	−0.494 b	0.621
AST (U/L)	64.37 ± 121.93	62.25 ± 110.21	83.30 ± 200.76	−0.342 b	0.732
ESR (mm/h)	60.82 ± 24.17	59.93 ± 23.81	69.29 ± 26.56	2.873^a^	0.092
CRP (mg/L)	89.43 ± 60.73	89.50 ± 61.67	88.79 ± 53.01	−0.229^b^	0.819
Platelet (× 10^9^/L)	377.25 ± 106.77	374.90 ± 107.22	397.56 ± 102.61	0.998 a	0.319
Total serum protein (g/L)	62.07 ± 8.03	62.02 ± 8.08	62.54 ± 7.77	0.088 a	0.767
Serum albumin (g/L)	34.36 ± 4.48	34.59 ± 4.43	32.30 ± 4.52	5.749 a	0.017
PNI	53.22 ± 10.11	54.06 ± 9.68	46.01 ± 11.10	15.010 a	<0.001

CALs, coronary artery lesions; PNI, prognostic nutritional index; CRP, C-reactive protein, ESR, erythrocyte sedimentation rate; ALT, alanine aminotransferase; AST, aspartate aminotransferase.

a Analysis of Variance (ANOVA).

b Mann-Whitney U test.

### Correlation between PNI and clinical parameters

The Prognostic Nutritional Index (PNI) was found to be positively correlated with leukocyte count (*r* = 0.329, *p* < 0.001) and platelet count (*r* = 0.309, *p* < 0.001). Conversely, PNI was negatively correlated with coronary artery diameter (*r* = −0.260, *p* < 0.001), age (*r* = −0.340, *p* < 0.001), C-reactive protein (CRP) levels (*r* = −0.208, *p* = 0.001), erythrocyte sedimentation rate (ESR) (*r* = −0.150, *p* = 0.025), total bilirubin (*r* = −0.214, *p* = 0.001), alanine aminotransferase (ALT) (*r* = −0.199, *p* = 0.003), and aspartate aminotransferase (AST) (*r* = −0.133, *p* = 0.041). No significant correlation was observed between PNI and neutrophil count or hemoglobin levels (Table 2).

**Table 2:** Correlations between the PNI and clinical parameters.

Variables	r	*P* value
Age	−0.340	<0.001
Internal coronary artery diameter	−0.260	<0.001
CRP	−0.208	0.001
ESR	−0.150	0.025
Leukocytes	0.329	<0.001
Neutrophils	−0.083	0.197
Platelets	0.309	<0.001
Hemoglobin	0.084	0.192
Total bilirubin	−0.214	0.001
ALT	−0.199	0.003
AST	−0.133	0.041

PNI, prognostic nutritional index; CRP, C-reactive protein, ESR, erythrocyte sedimentation rate; ALT, alanine aminotransferase; AST, aspartate aminotransferase.

### Diagnostic value of PNI in CALs

**Figure 1:**
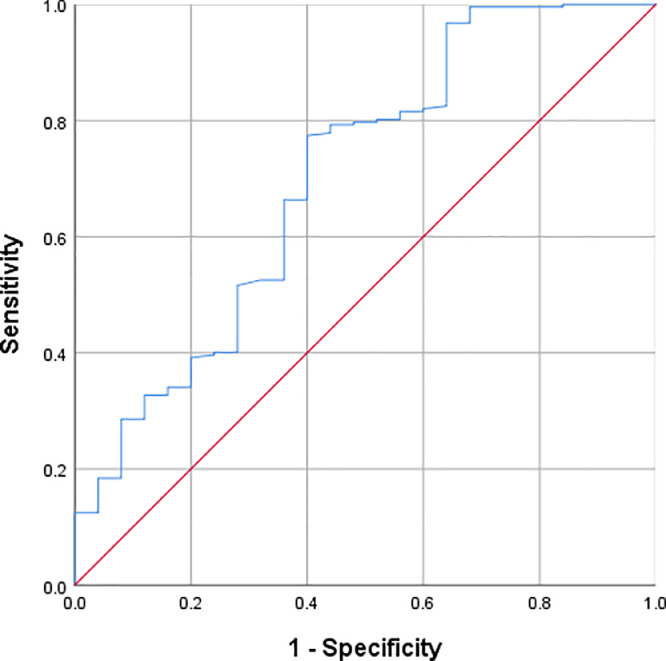
Receiver operating characteristic (ROC) curve for PNI in assessing the presence of CALs in KD patients.

Receiver operating characteristic (ROC) curve analysis was performed to assess the diagnostic value of PNI in identifying coronary artery lesions (CALs). The area under the ROC curve (AUC) for PNI in predicting CALs was 0.702 (95 % CI: 0.584–0.821, *p* = 0.001). The optimal cut-off value for PNI was determined to be 46.575, based on the maximum Youden index (0.374), with a sensitivity of 77 % and a specificity of 60 % (Figure 1).

### Association of PNI with the presence of CALs

Binary logistic regression analysis was conducted to identify independent risk factors for the presence of coronary artery lesions (CALs) among variables that demonstrated a significance level of *p* < 0.25 in univariate analyses. These variables included age, hemoglobin, lymphocyte count, erythrocyte sedimentation rate (ESR), serum albumin, and the Prognostic Nutritional Index (PNI). The results indicated that PNI was an independent protective factor against the presence of CALs, with an odds ratio (OR) of 0.916 (95 % confidence interval [CI]: 0.859 - 0.976, *p* = 0.007). Patients were categorized into high and low PNI groups based on the optimal cut-off value of 46.575. Further binary logistic regression analysis, treating PNI as a dichotomous variable, revealed that patients with low PNI had a 4.25-fold increased likelihood of having CALs compared to those with high PNI (OR = 4.25, 95 % CI: 1.688 - 10.697, *p* < 0.001). Detailed results are presented in Table 3, Table 4.

**Table 3:** Binary logistic regression analysis of factors influencing coronary artery lesions.

	B	Wald	*P* value	OR	95 % confidence interval of OR
PNI	−0.088	7.226	0.007	0.916	0.859–0.976

**Table 4:** Binary logistic regression analysis of factors influencing coronary artery lesions.

	B	Wald	*P* value	OR	95 % confidence interval of OR
High PNI patients (PNI ≥ 46.575)	1(reference)				
Low PNI patients (PNI<46.575)	−2.833	68.23	<0.001	4.250	1.688–10.697

## Discussion

This retrospective study investigated the association between the PNI and the presence of CALs in patients with KD. These findings indicate that higher PNI levels may serve as a protective factor against the development of CALs, with patients having low PNI levels (PNI < 46.575) being 4.25 times more likely to develop CALs compared to those with high PNI levels (PNI ≥ 46.575). These results highlight the potential clinical utility of PNI as a biomarker for identifying children at higher risk of CALs in KD.

The identification of risk factors for CALs is crucial for the early detection and management of KD, given that CALs are a significant cardiac complication and the leading cause of acquired heart disease in children in developed countries [1]. The present study found a 10.3 % incidence of CALs, which is consistent with previous reports [15,16]. However, the prevalence of coronary artery abnormalities observed was slightly lower than that reported in the 2004 AHA KD diagnostic guidelines [2]. This discrepancy may be attributed to the increased early treatment of KD patients since the publication of the 2004 guidelines, which might have contributed to the decreased incidence of CALs. Additionally, the hospital admits numerous patients with mild KD, a trend that was also observed during the study period.

PNI, derived from serum albumin and peripheral blood lymphocyte counts, provides a comprehensive overview of an individual's nutritional status and immune function and has increasingly emerged as a potential biomarker for disease prognosis assessment and treatment efficacy monitoring [17, 18, 19, 20]. The present study revealed significant differences in PNI across subgroups defined by CAL status, indicating PNI's potential role in influencing CALs. This finding is supported by previous research highlighting a strong correlation between PNI and the progression of coronary artery lesions in KD patients [12,21]. Lower PNI values have been shown to predict the development of coronary artery aneurysms (CAAs) and are an independent risk factor for enduring CAAs up to two years [12,21]. The study further validates these findings and demonstrates that PNI assessed within the first few days of fever onset may predict the presence of CALs more accurately.

The moderate diagnostic efficacy of PNI in identifying CALs in KD patients underscores its potential as a useful clinical tool. The use of PNI for diagnosing CALs demonstrated a sensitivity of 77 % and a specificity of 60 %, with an optimal cut-off value of 46.575 (AUC = 0.702, 95 % CI: 0.584 - 0.821, *p* = 0.001). These results suggest that PNI can be used as a biomarker to identify children at higher risk of developing CALs. Furthermore, the present study found that PNI levels were significantly lower in children with more severe coronary artery dilation, indicating that PNI may also serve as an important indicator of disease severity.

These findings are consistent with several previous studies. For instance, Yalcinkaya et al. [22]. found that lower PNI levels were independently associated with CALs in KD patients. Their study showed that PNI values in the CALs group were significantly lower than those in the group without CALs (*p* < 0.001). The present study further supports these findings by demonstrating a negative correlation between PNI and coronary artery diameter (*p* < 0.001). Additionally, Zhong et al [23]. conducted a systematic evaluation and meta-analysis, suggesting that PNI could be used as a biomarker to identify KD patients with CALs and those with IVIG resistance. The present study validates these findings and provides further evidence for the clinical value of PNI in early diagnosis.

This study has several limitations. Firstly, it was a single-center retrospective study with a potentially under-representative sample, which may limit the external validity of the results. Future studies should be conducted in larger, multicenter cohorts to validate these findings. Secondly, the limited sample size may have introduced bias in the statistical analysis, potentially affecting the detection of important associations. Despite these limitations, the present study provides valuable preliminary evidence that PNI can be an important predictor in the management of KD patients.

Although the present study focused on the PNl Index, the authors recognize that acute-phase HDL-cholesterol decline has been associated with the severity of systemic vasculitis and coronary artery lesions in KD [24]. Because serial lipid measurements were not systematically obtained, the authors could not investigate this relationship directly. Future large-sample, multi-center, prospective studies should therefore incorporate both PNI and serial HDL-cholesterol measurements to clarify their relative prognostic contributions and to explore potential combined predictive models.

For patients with extremely low PNI (< 46.575), the authors recommend early multidisciplinary evaluation by pediatric cardiologists and nutritionists. In addition to standard IVIG and anti-inflammatory therapy, high-protein nutritional support and intensified echocardiographic surveillance at 2 weeks, 6 weeks, and 6 months after onset should be considered. If PNI remains markedly reduced, short-course systemic corticosteroids may be cautiously added to rapidly control inflammation. Prospective multicenter trials are nevertheless needed to confirm the safety and efficacy of these interventions in reducing coronary artery lesions.

## Conclusion

In summary, the present study suggests that PNI may be a valuable biomarker for identifying children at higher risk of developing CALs in KD. Higher PNI levels appear to be protective against CALs, while lower levels are associated with a higher likelihood of CALs. PNI may also serve as an important indicator of disease severity, helping to identify children who require more intensive monitoring and treatment. Future studies should aim to validate these findings and explore the potential clinical applications of PNI in KD management.

## Funding information

This research was supported by the Anhui Province Clinical Key Specialty Construction Project Fund.

## Ethics approval and consent to participate

The ethics committee of Fuyang People’s Hospital approved the study protocol (No. [2022]73). Written informed consent was obtained from all patients or their legal guardians before the use of their medical records in this study. The study was conducted in accordance with the Declaration of Helsinki.

## Availability of data and materials

The datasets used and analyzed during the current study are available from the corresponding author on reasonable request.

## Conflicts of interest

The authors declare that they have no known competing financial interests or personal relationships that could have appeared to influence the work reported in this paper.
